# Patients on the psychosis spectrum employ an alternate brain network to engage in complex decision-making

**DOI:** 10.1371/journal.pone.0238774

**Published:** 2020-09-11

**Authors:** Kanchna Ramchandran, Jess Fiedorowicz, Zhaoying Chen, Yilin Bu, Antoine Bechara, Nancy C. Andreasen

**Affiliations:** 1 University of Iowa, Iowa City, Iowa, United States of America; 2 University of Ottawa, Ottawa, Canada; 3 The Ottawa Hospital, Ottawa, Canada; 4 Boston University, Boston, Massachusetts, United States of America; 5 University of Southern California, Los Angeles, California, United States of America; Texas A&M University, UNITED STATES

## Abstract

Brain reward processing mechanisms that underlie complex decision-making are compromised in psychosis. The goal of this research was to advance our understanding of the underlying (1) neural mechanisms and (2) discrete neuro-economic/motivational processes that may be altered in complex decision-making in euthymic patients on the psychosis spectrum (PPS). Utilizing a functional magnetic resonance neuroimaging (fmri) paradigm of a well-validated laboratory measure of complex decision-making (Iowa Gambling Task-IGT), the brain activation patterns of a target group of PPS were compared to a demographically matched healthy comparison group (HMC). These two groups were also evaluated on real-life decision outcomes on day of scan. PPS primarily activate the Dorsal Attentional Network (DAN) in real-life decision outcomes and in achieving similar levels of performance on the IGT as the HMC, in-spite of dysregulated dopamine-based brain-reward circuit and salience network fmri activation patterns. However, PPS report more significant negative outcomes of their decision-making in real-life, compared to HMC. The differential engagement of brain networks by PPS on the IGT appear to be moderated by antipsychotic, dopamine antagonist, medication lifetime/daily dose levels. These findings may also be mediated by extent of dysregulation in brain reward circuitry and salience network associated with psychosis severity in the target PPS group. This is also evident in case studies of unmedicated PPS. We conclude by suggesting that the brain may adapt to this dysregulation by co-opting the DAN network, which is implicated in the related function of problem-solving, towards complex decision-making. The extent of utilization of the DAN network in complex decision-making may be moderated by psychosis severity.

## Introduction

The explosive growth in basic neuroscientific research in the past decades has provided enormous clarity in the underlying neurobiology of dopamine-based reward processing mechanisms in the brain [1] and there is fair consensus in the literature identifying the components of reward processing in health and disease [2, 3], in complex decision-making(CDM) [4–6]. These include reward valuation/prediction and expectancy that drives selection or subjective choice; and initial or extended responsiveness to reward attainment (Win); generation of a reward prediction error signal in outcome processing of Lose; and updating mechanisms for adaptive reward learning.

While psychiatric research grows exponentially, its clinical impact is leveling off, since categorical approaches to nosology have failed to identify explanatory biological mechanisms in psychiatric disorders [7, 8]. However, there has been progress to our understanding of symptomatic dysregulation of brain reward processing circuitry in psychiatric conditions in reward processing [9–11], especially in the striatum and the prefrontal cortex. The dopamine hypotheses of psychosis posit that dysregulation in dopamine-based reward circuit mechanisms lead to psychotic symptoms involving (1) subcortical hyperdopaminergia (dopamine overdrive) (2) prefrontal hypodopaminergia (hypo-frontality) compromising top-down cognitive control networks; and (3) aberrant salience attribution (“the third dopamine hypothesis”) [12] additionally involving dysregulation of the salience network as well [13].

The goal of this research was to use a functional magnetic resonance neuroimaging (fmri) task as an indirect proxy to capture in vivo dysregulation of dopamine- based, reward processing components of CDM: subjective Choice (selection); outcome processing of initial/extended reward attainment (Win) or Lose (reward prediction error signal); and adaptive updating/ reward learning. This would permit us to indirectly evaluate cortico-striatal reward and salience processing dysregulation in psychosis, with the acknowledgment that fmri methods cannot directly assay neurotransmitter function. However, the blood oxygenation level dependent (BOLD) response in event -related design of fmri would allow us to view in vivo, real-time activity of cortico-striatal reward and salience networks as they engage in the distinct components of reward-based CDM.

Hence, with an experimental task, we examined dysregulations in CDM in euthymic, psychiatric patients who shared a common symptom pattern: the experience of psychosis. Practically speaking, they were individuals who had been assigned DSM-IV or V [14] diagnoses such as disorders in the schizophrenia spectrum and the mood spectrum with psychotic features. We ignored their various categorical diagnoses and instead treated these patients as a single group of psychosis spectrum patients (PPS), since we were interested in the over-arching neurobiology underlying reward and salience processing dysregulation in psychosis.

Thus, we measured the severity of psychotic symptoms in a group of remitted, euthymic PPS as continuous dimensions, along with their antipsychotic medication dosage; and related them to their reward brain circuit dysfunction captured in fmri of their performance on a reliable, ecologically valid CDM task (Iowa Gambling Task-IGT [15, 16]). We compared their fmri performance on the IGT in separate stages of CDM (Choice, Win, Lose and updating/learning) with that of a demographically matched healthy comparison (HMC) group of individuals. We also compared their (PPS, HMC) fmri activation patterns (each stage of IGT-Choice, Win, Lose, Updating/learning) co-varied with IGT score; as well as with the IGT fmri activation patterns co-varied with scores on a real-life Decision Outcome Inventory (DOI), in order to better assay ecological validity of CDM. Please find the list of all fmri activation patterns in this study in the S1 Table. The data used in this study is available in S1 Data.

The aim of this research was to parcellate and correlate bottom-up sub-cortical processing with top-down regulation of cortical control networks with specific components of reward processing in CDM. And to examine how these may be compromised in psychosis as an indirect assay of dopamine dysregulation. We predicted that that PPS would demonstrate impaired performance on the IGT compared to the HMC as has been well established [17]. This may be due to dysregulation of the more medially oriented brain reward circuit [1] due to hypo frontality of the medial prefrontal cortex (mPFC), associated with reward-related subjective choice. However, PPS activated the laterally oriented dorsal attentional network (DAN) [18], a key system of working memory, executive control and problem-solving, in all components of CDM achieving the same level of performance on the IGT as the HMC. We discuss these fmri results in the light of the dopamine hypotheses of psychosis, attempting to tease out the relative roles of disease versus antipsychotic medication burden that might account for these results. We also explored the real-life implications of this dysregulation in the context of decision outcomes.

## Results and discussion

The goal of this study was to examine CDM and brain reward and salience network circuitry dysregulation in patients on the psychosis spectrum. It is therefore of note that PSS and HMC groups perform at the same level on the IGT, there being no significant difference in the raw IGT outcome score (t = -1.054, df = 31, p-value = 0.3) or money earned (t = 0.842, df = 29, p-value = 0.4) between the two groups (see Methods section for task details). This may be due to the fact that the K’L’M’N’ version, used in this study, has only 80 trials than the more commonly used A’B’C’D’ version which has 100 trials [19] and hence all participants have fewer trials than the A’B’C’D’ version in establishing their learning curves in distinguishing between good and bad decks. The K’L’M’N’ version is also more challenging than the A’B’C’D version and while robust [19] may have floor effects in distinguishing between advantageous and disadvantageous selections, especially in the earlier, learning blocks of trials, that may be impacting final score, as has been observed in another fmri study [20]. However, the pattern of BOLD response on the IGT in the two groups, may not be secondary to their IGT score and may help distinguish the pattern of their CDM performance.

**Fig 1:**
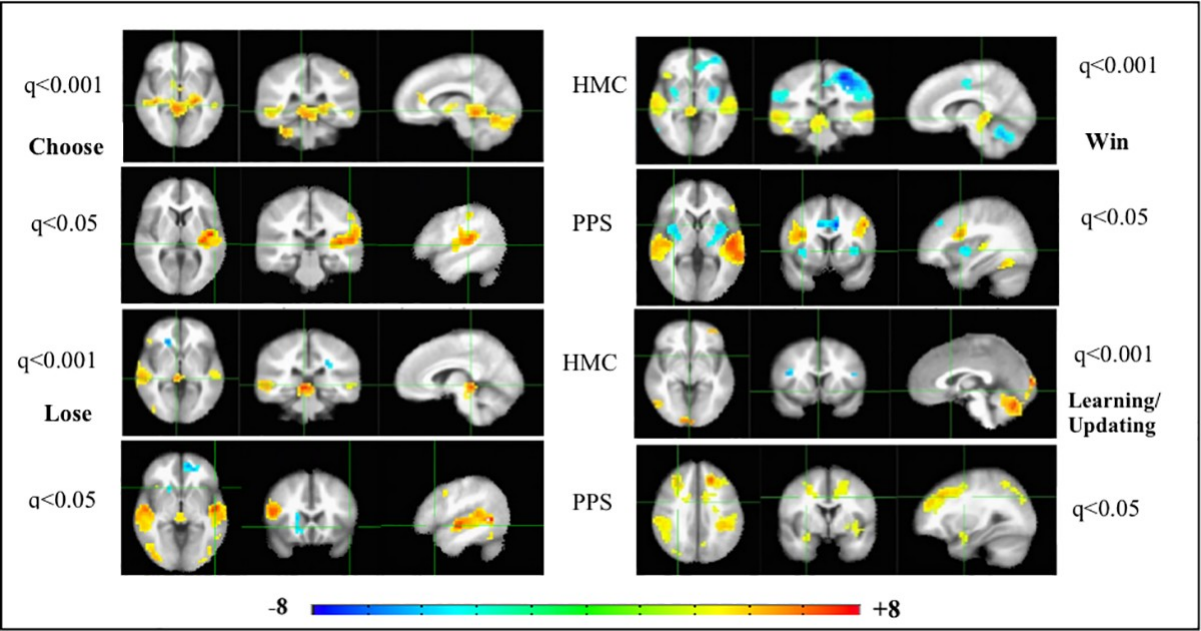
Functional neuroimaging activation patterns of the Iowa Gambling Task in patients on the psychosis spectrum and healthy individuals. Left to right are the axial, coronal and sagittal views of the significant t-statistic, averaged across all psychosis spectrum patients (PPS), or healthy matched comparison (HMC) individuals, for each of the 4 conditions in the Iowa Gambling Task (IGT). Multiple comparisons corrected for False Discovery Rate (FDR-q).

The BOLD response on the IGT conditions (Fig 1, see methods section for details of fmri task conditions/events) however, indicate significantly different activation patterns in the two groups of HMC and PPS. During subjective choice (card selection -Choose event), HMC activate the expected brain reward circuit [21, 22] extending from the regions of cerebellum [23], midbrain dopamine (DA) region, thalamus, dorsomedial (caudate) and ventral striatum, and mPFC. HMC also activate the left dorsolateral prefrontal cortex (l-dlPFC). PPS (Fig 1, Choose), show no significant activation of the brain reward circuit. Instead PPS significantly activate key DAN nodes of l-dlPFC and left superior parietal lobule (l-SPL); and left Insula (l-Ins), a key node of the salience network [13].

Both HMC and PPS activate the temporal cortex (Fig 1, Choose): HMC activate the bilateral middle temporal gyrus (bi-MTG), while the PPS activate the left superior temporal gyrus (l-STG) especially the Heschyl’s gyrus (auditory cortex). The activation of the temporal gyrus remains a constant theme for all events of Choose, and outcome processing events of Win and Lose for both HMC and PPS. The temporal gyrus, especially the STG are bi-directionally connected with the key areas such as insular, primary sensory and their association cortices that serve as “somatic markers”: carrying affective, sensory and visceral information that provide salient cues of reward and loss between the brain and body [13, 24, 25].

Outcome processing of reward attainment (Win) activates the midbrain DA region putatively signaling dopamine release in HMC (Fig 1). This coupled with activation of the right -orbital frontal cortex (r-OFC) and right-Ins, signal reward valuation/salience of a winning choice in that trial. These areas are not activated in PPS, who show significant de-activation of the bi-Ins, even after winning money in a trial. This is borne out by anecdotal evidence of PPS never expressing/demonstrating any positive affect/feedback to research staff on winning money or succeeding, unlike the HMC who were usually happy to win money in the game.

Outcome processing of Lose feedback in a trial (Fig 1, Lose) should putatively trigger the generation of reward prediction error (RPE) signal in midbrain DA neurons [26] and we see strong activity in this region, in both HMC and PPS. This is accompanied by frontal activity in r-OFC in HMC, presumably associated with reward valuation signaling. In PPS, we see frontal activation of the bi-dlFC, with hypo-frontality (mPFC-deactivation) associated with the dopamine hypothesis of psychosis [27]. Thus, in PPS, even though RPE signaling may occur in response to Lose feedback, it does not appear to engage the rest of the brain reward circuit; leading to the possibility that any top-down regulation on identifying patterns of losing card selections (bad decks) and subsequent cognitive control of avoidance of those decks may involve engaging the dlPFC node of DAN [28].

This is further bolstered by the significant activation in PPS of the DAN network nodes of bi-dlFC, bi-SPL/precuneus during the block of updating/learning task condition (Fig 1). This is accompanied by activation of the bi-Para hippocampal gyrus in PPS. None of these regions are activated in HMC during the learning/updating task block, which show activity in l-OFC, cerebellum and occipital cortex. It is possible that adaptive reward learning before the onset of the next trial, occurs during reward valuation/RPE signaling in the brain reward circuit (associative/habit learning circuits [21]) during outcome processing in healthy brains. PPS on the other hand, due to dysregulation of the brain reward circuit, may be slower to respond, and co-opt the DAN and declarative memory networks to achieve the same end, during the learning/updating phase. We also note that it appears that healthy brains have overall higher activation levels in all task conditions, with the BOLD response surviving FDR correction of q<0.001; while the BOLD response in PPS could survive FDR correction of only q<0.05. This could indicate that whole brain activity and percent signal change in healthy brains in response to the IGT was much higher than that of PPS.

**Fig 2:**
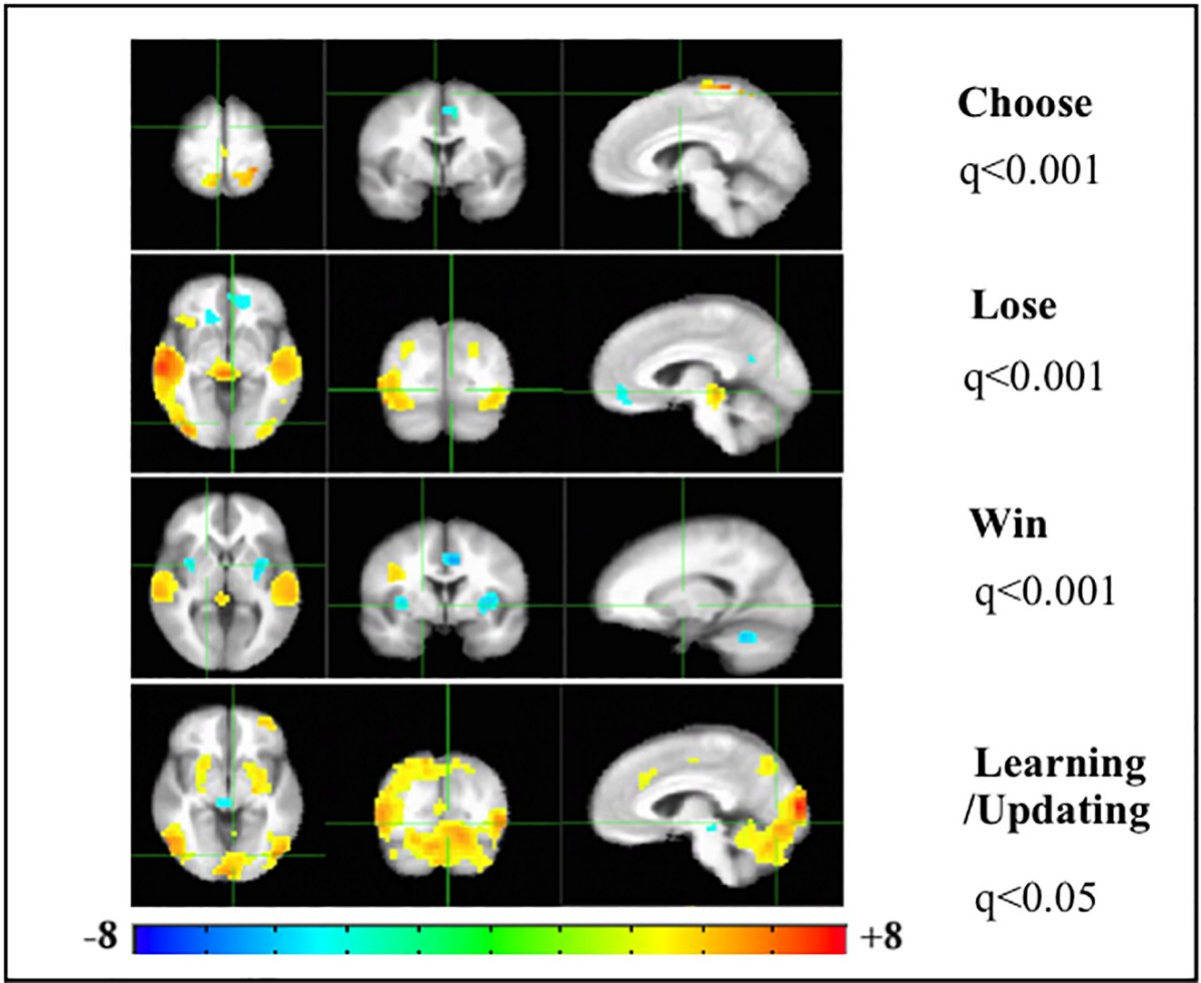
Analysis of *relative* functional neuroimaging activation patterns in patients on the psychosis spectrum and healthy individuals on the Iowa Gambling Task. Analysis of Co-variance (ANCOVA), of mental health status with each of the 4 conditions in the Iowa Gambling Task (IGT). Mental health status was coded as 1 for each patient on the psychosis spectrum (PPS) and as 0 for each healthy matched comparison (HMC) individual with a total group N = 34, comprising of 17 PPS and 17HMC. Left to right are the axial, coronal and sagittal views of the significant t-statistic of the group activation patterns. Multiple comparisons corrected for False Discovery Rate (FDR-q). Hot/cool colors indicate greater activation by PPS/HMC and vice-versa (Cool/Hot colors indicate lesser activation by PPS/HMC).

We delved into these results further by examining which brain regions were relatively higher/lower in activation for the two HMC and PPS groups by combining both groups in the same analytic model by coding them on mental health status as (1) PPS and (0) healthy-HMC. Thus, in Fig 2, activation patterns would be diametrically opposite in HMC and PPS (i.e.) warm colors would be associated with higher activation in PPS (coded with the higher number -1) and cool colors would be associated with higher activation in HMC (coded with the lower number -0). And vice versa- meaning that cool colors would in indicate regions with lower activation in PPS and warm colors would indicate lower activation in HMC.

The top panel of Fig 2 indicates that the bi-SPL/precuneus nodes of DAN are activated more in PPS while the mPFC of the brain reward circuit is activated more in HMC during subjective choice (card selection) in the IGT. This also indicates hypo-frontality in PPS during subjective choice, further corroborating our previously discussed results from Fig 1.

The Lose panel indicates that contrary to expectation PPS have higher RPE signaling in midbrain DA regions than HMC in response to loss, including higher activation of anterior r-Ins and r-OFC, involved with reward valuation of salient choice in deck selection. However, with hypo frontality of the mPFC of the brain reward circuit, PPS engage with the r-OFC and bi-SPL (higher activation) to exercise cognitive control networks for future avoidance of bad decks.

In the Win condition too, PPS have higher signaling from midbrain DA regions than HMC, but show greater hypo frontality in mPFC, perhaps co-opting the r-dlPFC node of DAN instead in reward valuation. The PPS’ lowered activation of the bi-Ins implicated in visceral feeling [24, 29] than HMC, in response to winning money, is perhaps associated with anhedonia so characteristic of psychosis [9, 30].

The higher activation of midbrain DA regions in outcome processing (Win and Lose) in PPS maybe putatively linked excessive/chaotic release of dopamine. This may lend aberrant assignment of salience to innocuous stimuli: the chaotic release of dopamine, sometimes in the context of trivial everyday events, produces an attribution of excessive/hyper incentive salience to these stimuli, resulting in psychotic symptoms [31]. The hypo frontality of the mPFC and the deactivation of the bi-Ins (Win), both key nodes of the salience network [13] further suggests that psychotic symptoms in PPS may be driven by this brain reward circuit dysregulation.

In reward learning/updating block of the IGT, PPS display higher activations in the lateral posterior and frontal nodes of DAN, than HMC; while healthy brains show higher signaling of midbrain DA brain regions. This second analysis displayed in Fig 2 corroborates the results from Fig 1, in the differential engagement of two brain networks in reward based CDM in healthy people (brain reward circuit) and PPS (DAN).

### Antipsychotic medication effects on brain reward circuit dysregulation and psychosis

**Fig 3:**
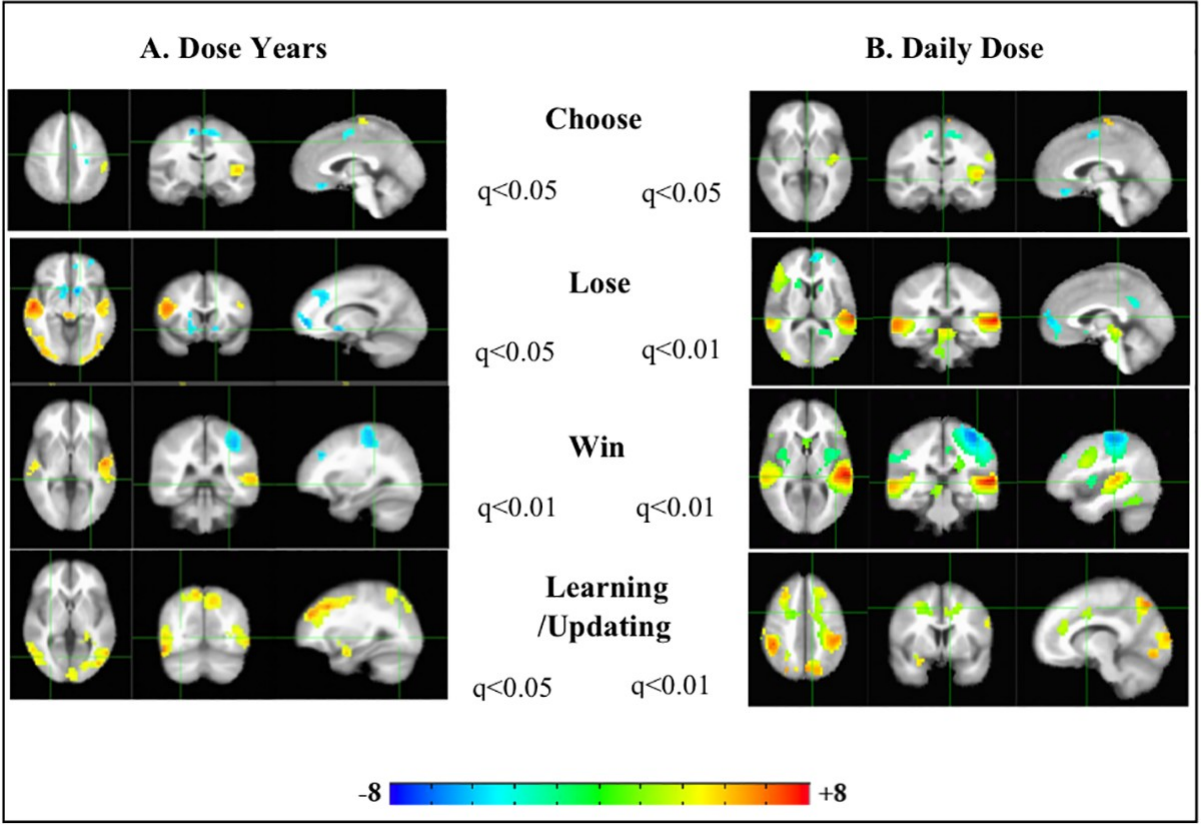
Moderation of functional neuroimaging activation patterns in patients on the psychosis spectrum by antipsychotic medication dosage on the Iowa Gambling Task. Analysis of Co-variance (ANCOVA), of antipsychotic medication lifetime dosage (PANEL A), or dose on day of functional neuroimaging (fmri) scan (Panel B) for each patient, with the 4 conditions in the Iowa Gambling Task (IGT). N of patients on the psychosis spectrum (PPS) = 17. Left to right (within Panels A and B) are the axial, coronal and sagittal views of the significant t-statistic of the group activation patterns. Multiple comparisons corrected for False Discovery Rate (FDR-q). Hot/cool colors indicate greater/lesser medication dose load.

Version I of the “dopamine hypothesis” arose in the 1960s-1970s, when it was recognized that the effectiveness of antipsychotic medications was tightly linked to their affinity for dopamine D2 receptors, leading to the view that psychosis was “caused” by excessive activity in the dopamine system and reduced via D2 blockade [31–34]. Hence, we examined the role of life-time and daily (day of scan) dosage of antipsychotic medication prescriptions in activation of brain reward circuitry [35] in CDM. We included antipsychotic dose as a covariate with the activation patterns for each condition on the IGT (Fig 3), to check if the DA receptor antagonism of these medications were driving the activation patterns in PPS on each of the IGT conditions.

The activation pattern of antipsychotic medication dosage on day of scan follows the general pattern of the PPS’ lifetime dose years across task conditions (Fig 3) suggesting that cumulative medication dose load has a consistent trend over time on how it may be impacting brain reward circuit function in CDM. We see hypo frontality in mPFC and lowered activation in striatum (dose years/daily dose) and higher activity in midbrain DA region (daily dose) amongst patients with higher medication load and vice versa (those with lowered medication load, would activate these regions more) (Fig 3) across the various task conditions. We also see greater activation in the DAN nodes of r-dlPFC (Lose-Dose years), bi-dlPFC/ l-Para hippocampal gyrus in (Dose years/ Daily Dose years: Learning/Updating) and bi-SPL (Dose years/ Daily dose in Choose/Updating/Learning) (Fig 3), suggesting that this network may be recruited in CDM for top-down regulation.

The question is raised however, if antipsychotic medication dose load merely correlates directly with the severity of psychosis symptoms that it is meant to manage [36]. Hence, we examined the activation patterns on the IGT of the only two patients in the PPS who had been medication free for more than 5 years seen in S1 Fig, which may be viewed in the S1 File. These case studies suggest that the presence of psychosis symptoms may additionally impact dysregulation of brain reward circuity in PPS, along with antipsychotic medication dose load. Details of these case studies may be found at S1 File.

### Psychosis symptoms and brain reward circuit activation in CDM

The “dopamine hypothesis” involving brain reward circuitry [37] was formulated, when PET imaging studies permitted investigators to visualize cerebral blood flow and metabolism and to measure the activity of dopamine receptors *in* vivo [10, 11, 38]. The recognition that D1 receptors are localized in the prefrontal cortex, while D2 receptors are striatal, when combined with the mounting evidence for “hypo frontality” in schizophrenia, led to the proposal that the fundamental disruption was due to hypodopaminergia in prefrontal cortex leading to a failure in top-down regulation of the striatum and a resultant striatal hyperdopaminergia [27]. These two hypotheses emphasized the role of dopamine in psychosis. Version III, proposed by Kapur [31], made an innovative connection between the study of dopamine and brain reward processing to provide an explanation as to how increased dopaminergic tone could lead to the clinical picture of psychosis using the concepts of salience and reward. The suggestion was that the chaotic release of dopamine, sometimes in the context of trivial everyday events, produces an attribution of excessive/hyper or diminished/hypo incentive salience to innocuous stimuli. This may be due to dysregulation in the salience network (comprising of the key nodes of anterior insula and dorsal anterior cingulate/medial prefrontal cortex) [31].

**Fig 4:**
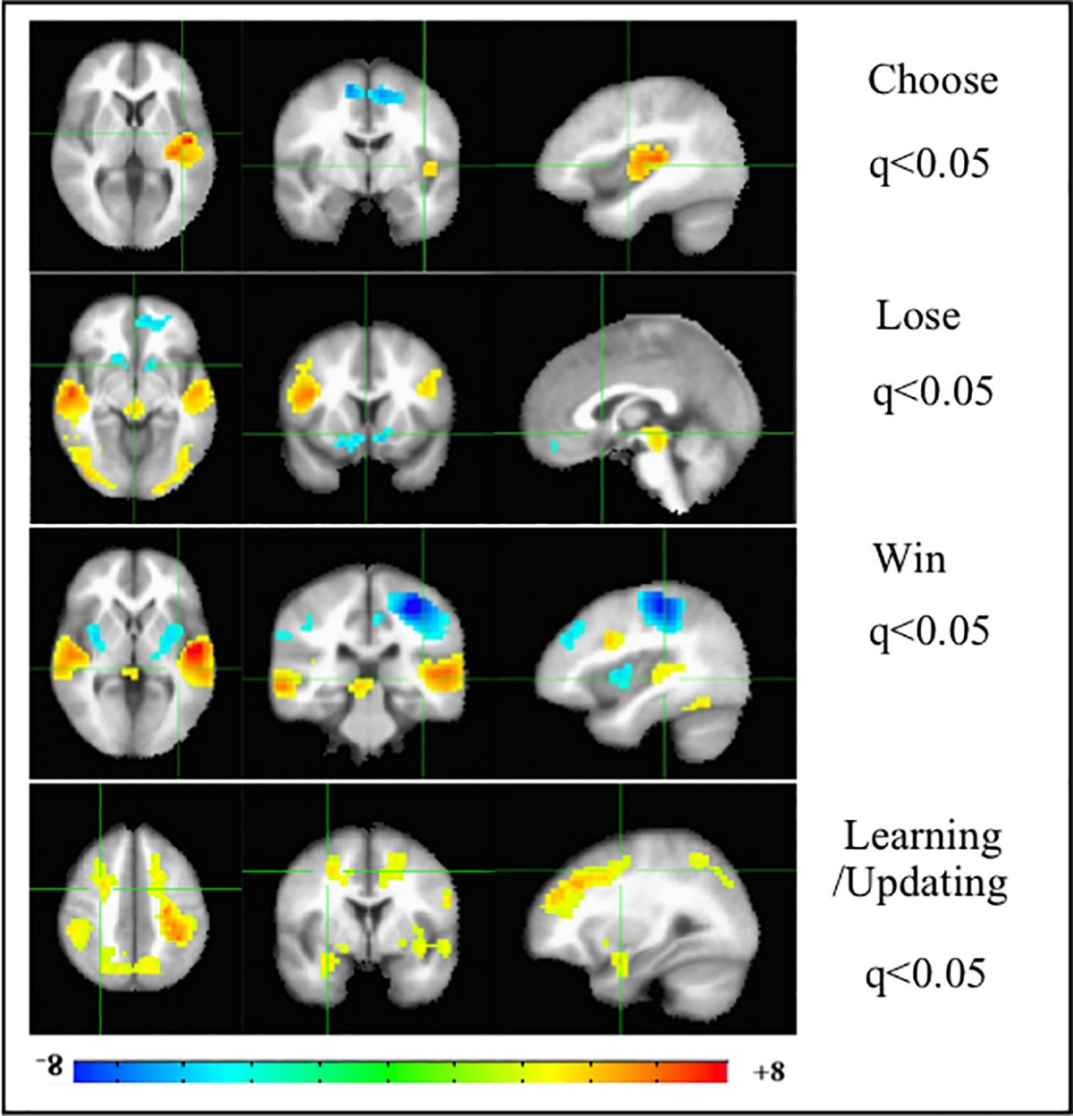
Moderation of the functional neuroimaging activation patterns on the Iowa Gambling Task by psychosis symptoms in patients on the psychosis spectrum. Analysis of Co-variance (ANCOVA), of total psychosis symptom score (positive + negative symptoms for each patient) with the 4 conditions in the Iowa Gambling Task (IGT). N of patients on the psychosis spectrum (PPS) = 17. Left to right are the axial, coronal and sagittal views of the significant t-statistic of the group activation patterns. Multiple comparisons corrected for False Discovery Rate (FDR-q). Hot/cool colors indicate greater/lesser total psychosis symptom score.

Hence we co-varied the total sum of psychosis symptoms (positive + negative) measured on the day of scan, with the BOLD response on each of the IGT task conditions, such that warm colors would denote greater/lower activation by those PPS with higher/lower total psychosis score on the PANSS, and cooler colors would denote higher/lower activation patterns by those PPS with lower/higher total psychosis scores (Fig 4).

Irrespective of whether the PPS have higher or lower psychosis scores, one of the 2 salience network nodes (mPFC and l-Ins) is inhibited while the other is significantly activated in the Choose IGT condition. Thus, all PPS show dysregulation of the key nodes of the salience network [39] during subjective choice in the IGT.

During outcome processing (Win/Lose), irrespective of whether the PPS have lower or higher psychosis symptom score, we see dysregulation of the two ends of brain reward circuit, mPFC and midbrain DA regions, with one node activated if the other is deactivated or vice versa. Thus, all PPS have dysregulated brain reward circuit during reward/punishment feedback. Those PPS with higher psychosis symptom scores activate the bi-dlPFC node of DAN, during outcome processing. Outcome processing is the more likely phase of complex decision-making when an RPE signal is generated, and the significant activations of the bilateral temporal cortex/Heschyl’s gyrus and the association visual cortex (including fusiform gyrus), may be implicated in increased positive symptoms such as auditory and visual hallucinations.

During the learning/updating task block, PPS with higher psychosis symptomology activate the bilateral DAN nodes of dlFC and SPL/precuneus and para-hippocampal gyrus. These fmri results provide further support to Kapur’s 3^rd^ and updated version of the dopamine hypothesis of schizophrenia [31] involving dysregulated salience networks. Our fmri findings in this PPS symptoms analysis provides evidence of dysregulation of (1) the salience network, (2) the brain reward circuit with the (3) substitution effect of the dorsal attentional network in psychosis, during complex decision-making.

### Ecological validity in complex decision-making

**Fig 5:**
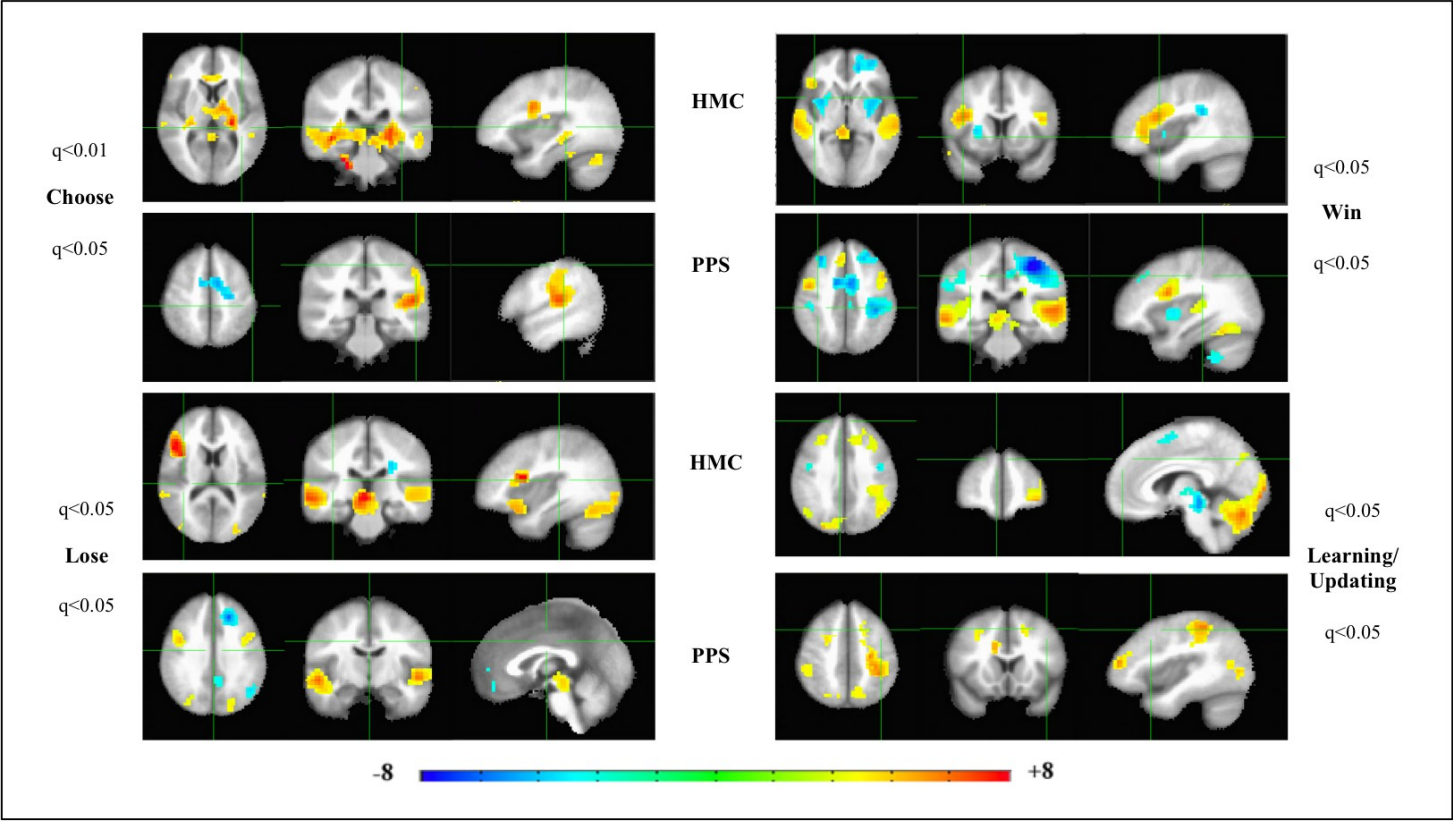
Moderation of the functional neuroimaging activation patterns on the Iowa Gambling Task by their IGT scores in patients on the psychosis spectrum and healthy individuals. Analysis of Co-variance (ANCOVA), of total IGT score with the 4 fmri conditions in the Iowa Gambling Task (IGT). N of patients on the psychosis spectrum (PPS) = 17, healthy matched comparisons (N) = 17. Left to right are the axial, coronal and sagittal views of the significant t-statistic of each group’s activation patterns. Multiple comparisons corrected for False Discovery Rate (FDR-q). Hot/cool colors indicate higher/lower total IGT score.

While there was no significant difference between PPS and HMC on IGT raw score (as reported in earlier in this study) within a fmri environment, we co-varied their IGT scores in each of the conditions of Choice, Lose, Win and Learning/updating, to examine activation patterns of those who performed better on the IGT versus (hot colors) those did not (cool colors). These results may be viewed in Fig 5.

It is of note that the activation patterns of Fig 5, with IGT score co-varied, essentially replicates Fig 1, which contains just the activation patterns on each of the 4 conditions of the IGT, in both PPS and HMC. This lends further credence to our results that those PPS with higher IGT score utilize the DAN network to problem -solve on the IGT in lieu of the brain reward circuit. The key difference seems to lie in the left anterior insula being activated in PPS who score higher on the IGT, when they are confronted with information of loss, suggesting higher somatic marker activity in these patients.

While the above results track brain reward circuit function *in vivo* through fmri, we examined how this may be translated in real-life behavior. PPS experienced significantly greater and more negative real-world decision outcomes (lower mean score reflects worse negative outcomes-Table 1), than HMC, as measured on the Decision Outcome Inventory (DOI) [40] (Table 1). The DOI is a self-report instrument that provides a score of avoidance of real-life outcomes that could have resulted by poor decisions that the respondent made in real life. The outcomes could be serious (example, bankruptcy) ranging to minor such as paying a library fine.

**Table 1:** Significant differences between patients on the psychosis spectrum and healthy matched individuals on behavioral measures of real-life outcomes of decision-making: Significant t-statistics on the Decision Outcome Inventory (DOI).

SCORE	Decision Outcome Inventory
Group Mean for PPS (N = 17)	23.125
Group Mean for HMC(N = 17)	28.611
t-statistic	-2.299
*p* value (two-tail)	0.029

**Fig 6:**
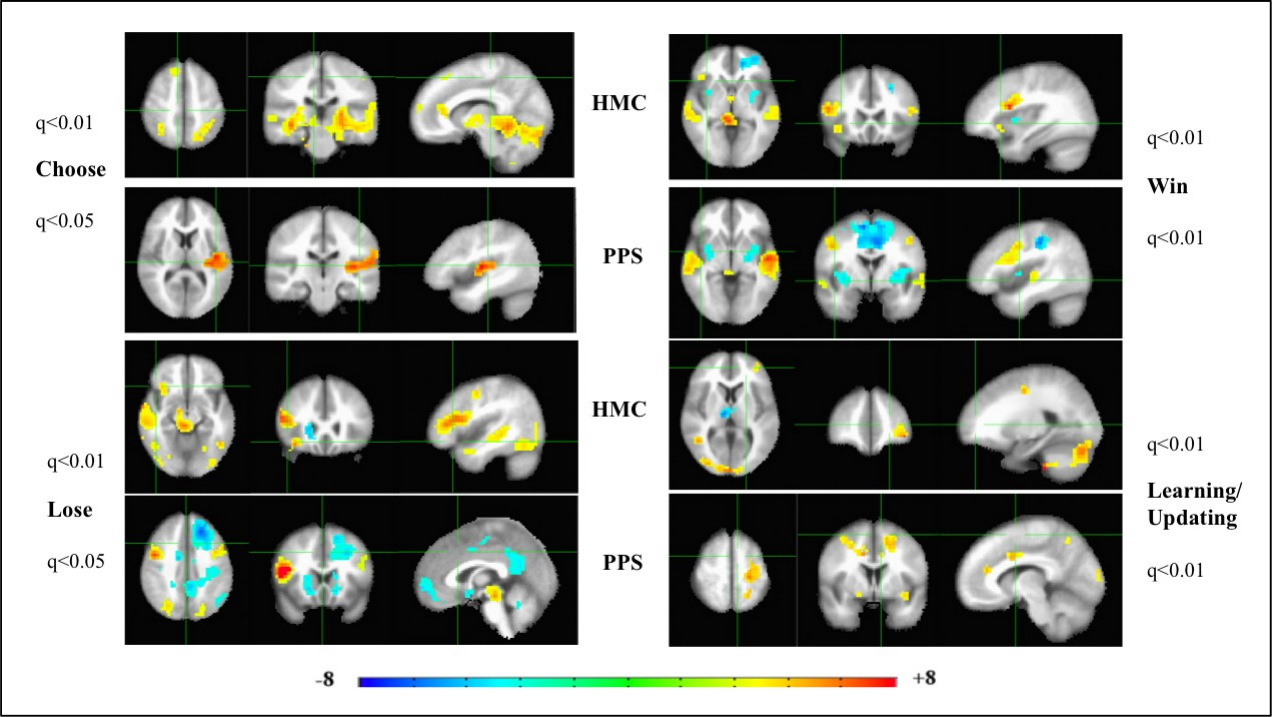
Moderation of the functional neuroimaging activation patterns on the Iowa Gambling Task by their Decision Outcome Inventory (DOI) scores in patients on the psychosis spectrum patients and healthy individuals. Analysis of Co-variance (ANCOVA), of total DOI score with the 4 fmri conditions in the Iowa Gambling Task (IGT). N of patients on the psychosis spectrum (PPS) = 17, healthy matched comparisons (N) = 17. Left to right are the axial, coronal and sagittal views of the significant t-statistic of each group’s activation patterns. Multiple comparisons corrected for False Discovery Rate (FDR-q). Hot/cool colors indicate higher/lower total DOI score.

We co-varied the DOI score on to each of the 4 conditions of the IGT fmri task and examined the resultant neural activation patterns in Fig 6.

It is of note that the fmri activation patterns co-varied with DOI score (Fig 6) are very similar to that of co-varied with IGT score (Fig 5), strengthening the ecological validity of the IGT task in predicting real-life CDM with PPS activating the DAN network and HMC activating the brain reward circuit in those participants who successfully minimized negative real-life decision outcomes. However, there is one notable exception in Fig 6. Those PPS with higher DOI score activate sections of the ventral striatum and the para-hippocampal gyrus in the last learning/updating phase of the IGT task, presumably encoding and updating information from previous trials, before the onset of the next trial. This is not visible in those PPS with higher IGT scores, during the learning/updating phase. Thus, those PPS who are better at avoiding real-like decision outcomes may have a better ability to encode salient experiential learning outcomes in memory.

A common theme in the fmri version of the IGT in Figs 5 and 6 are the activation of the posterior, temporal cortex, especially the auditory cortex in the Choice and Outcome (Win/Lose) processing events in both PPS and HMC, but more pronounced in PPS. While this region is a primary sensory cortex, it has bi-directional connections with the insula, primary and association sensory cortices, and perhaps serves as an integrated hub of somatic marker activity that informs CDM. Recent structural imaging research has suggested that the cortical thickness of the auditory cortex predicts IGT score, especially learning across blocks from start to finish in the task [41]. This research [41], tapped older adults who may be impaired in CDM, as would psychiatric patients, suggesting that the auditory cortex hub of somatic marker information may play a critical role in both brain reward and DAN networks in real-life and ecologically valid decision-making measures.

The dysregulation of the brain’s reward processing and salience networks with the co-opting of the DAN network [42], perhaps results in a patchwork of inconsistent behavioral modes that result in PPS facing higher negative outcomes of complex decision-making in real life, compared to healthy individuals.

While this study utilized a relatively small dataset, future research can tease out the complexities discussed above in larger samples. This would also allow more fine-grained analyses of activation patterns in the IGT correlated with psychosis symptoms and behavioral measures that may facilitate a neurobiological differentiation amongst categorically diagnosed PPS.

Thus, the impact of a future research effort could be to isolate trait measures and characteristics that capture essential differences in brain reward circuitry as they apply to differing forms of psychosis. This could potentially lead to customized treatment targets (anatomical, chemical and neurorehabilitation). The results of this pilot research reported in this study may contribute in future towards a new, viable nomenclature of distinguishing mental health syndromes from the perspective of dysregulation in preference-based, complex decision-making systems in the brain.

## Materials and methods

IRB ID:201204720, from the Institutional Review Board of the Human Subjects Office at the University of Iowa, has approved this study. All participants have read and signed the informed consent document for this research.

### Participant demographic description

We studied a community dwelling population of euthymic psychiatric patients (PPS) (N = 17), exhibiting symptoms of psychosis with one of the following diagnoses of schizophrenia, bipolar disorder or schizoaffective disorder. These PPS had an age range 26 to 56 years (Mean = 41.25 years, SD = 14.95); sex ratio of 64.7% females and education range of 12–22 years (Mean = 14.95, SD = 3.05). We also collected data on a healthy, demographically matched comparison (HMC) (N = 17) group, free of any psychiatric and neurological disease, age range of 26 to 61years (Mean = 47.35 years, SD = 12.72), sex ratios of 64.7% females and education ranging of 12–21 years (Mean = 17.25years, SD = 2.2). Participants were consented for research according to the institutional review board’s policy.

No participants were excluded from the analyses. Motion correction was a stage in fmri pre-processing and none had skewed levels that were high enough to warrant exclusion. Study wide exclusion criteria included individuals who were not MRI compatible and minors. Only euthymic patients were included since they needed to demonstrate enough stability in order to follow instructions and perform tasks in the MRI scanner. PPS were recruited from the Iowa Mood and Affect Research Registry and the Iowa Longitudinal Study on Schizophrenia Registry by advertisement. HMC were invited to participate in the study from a neuropsychologically profiled registry of healthy individuals, maintained by Dr. Ramchandran, based on their demographic matches to PPS.

#### Iowa Gambling Task

Each of these participants were administered the IGT, in the fmri scanner, an ecologically valid laboratory task of CDM [16] that involves the processing of reward and punishment, utilized in over 800 studies. The IGT measures decision-making under conditions of ambiguity and uncertainty and has a strong learning component. It captures the ability to learn from losses and to detect a pattern over time that rewards choices that result in incremental gains (accompanied by small losses) and punishes an emphasis on highly risky choices aimed toward large rewards. Each participant saw a screen on which were shown 4 decks of cards. The subject could select (click on using a manipulandum strapped to the hand) a card from any deck. On each choice, the face of the card appeared on top of the deck, and a message was displayed on the screen indicating the amount of money the subject had won or lost. At the top of the screen was a green bar that changed according to the amount of money won or lost after each selection. Once the money was added or subtracted, the face of the card disappeared, and the subject could select another card. The total number of trials was set at 80 card selections. Participants were instructed that they were free to switch from one deck to another at any time, and as often as they wished. The goal in the game was to win as much money as possible.

Total raw score (good decks minus bad) was the output score on the task, and the raw scores were used in the analysis. As the participant started sampling the decks, the feedback s/he received on reward and punishment provided clues for identifying good versus bad decks. Thus, ideally, participants learned over time that some of the decks were riskier choices (with large rewards yet crippling punishments), but that other decks accumulated financial gain in the long run (smaller rewards with smaller punishments). Thus, the task encouraged the participant to modify subjective choice and improve decision-making so that financial gain accrued over time. Task uncertainty simulated real life by providing no clues as to when the game might end. Further task description and details may be accessed at [15, 43, 44].

#### Scan parameters

Anatomical images were collected on a GE 750W scanner using a 32-channel head coil. Three-dimensional (3D) T1 weighted images (5mins.48secs) using a were collected using IR-prepped gradient echo spoiled grass sequence (BRAVO) in the coronal plane with the following parameters: TI = 450ms, TE = 3ms, TR = 8.5ms, flip angle = 10, FOV = 256x256x240mm, matrix = 256x256x240, bandwidth = 244Hz/pixel, acceleration = 2. 3D T2-weighted images (3mins 55secs) were collected using a variable flip angle fast spin-echo sequence (CUBE) with the following parameters: TE = 90ms, TR = 3000ms, echo train length = 130, FOV = 256x256x176mm, matrix = 256x256x176, bandwidth = 488Hz/pixel, acceleration = 2. The fmri data on the IGT (12mins.14secs) were acquired using a T2*-weighted echo planar imaging sequence (TR/TE = 2000/20ms, flip angle = 80°, FOV = 220mm; 35 axial slices, 2mm thick with no gap); The slices are acquired in an oblique, transverse orientation of approximately 23 degrees clockwise to the anterior/posterior commissure to minimize susceptibility artifacts for the mPFC. Additionally, during imaging data acquisition, each participant’s head was held within a static helmet-like device in the scanner, to minimize head motion.

The fmri protocol for the IGT was administered in the scanner as per a previously published protocol [20]. The K’L’M’N’ version of the IGT involving 80 trials is a reliable, validated version of the IGT and was administered to minimize practice effects [19, 45] since some of the participants had previous exposure to the A’B’C’D’ version. We contrasted the BOLD signal between 4 conditions using a quasi-event related design. From the start of any trial, there were three sequential events of (1) “Choose” (3-5secs) when participants clicked on the manipulandum to make a card selection; (2) “Win Feedback” (2 secs) for reward feedback on Winning money followed by, (3) “Lose Feedback” (2-5secs) when they received punishment feedback of money Lose that flashed on the screen. It is of note that the task was set up in such a way that the participant received a reward (“Win feedback”) in every trial, followed sequentially by a punishment (“Lose feedback”) in 25% of trials. The final 4^th^ condition “Updating/Learning”- a block design of 2-6s captured BOLD response between *Win Feedback* and start of next trial. The intention of this 4^th^ condition was to map activation patterns in the brain that may be associated with learning/updating accrued from current/previous trials that may inform decision-making and choice (card selection) in the upcoming trial. This 4^th^ condition occurred only in 75% of trials when “Lose Feedback” was absent, to maintain constant the total task time of 12mins14secs. Thus, the 4 task conditions were intended to capture key stages of the dopamine-based reward processing system in the brain [1, 21].

#### Data pre-processing, image and group analysis

This was conducted using the analysis of Functional Neuroimages [46] (AFNI) software for slice-time correction, de-spiking of random noise, motion correction, spatial smoothing and normalization. Filter size was set at three times of voxel size of 3.5mm distinguish functionally disparate regions while clustering homologous anatomical regions. The imaging raw data were batch pre/processed using the Andreasen lab’s automated pipeline that utilized the AFNI software’s robust [47], conventional, motion correction algorithms. The processing pipeline was set to flag any participant imaging data that exceeded the limits set for 3Tesla scanners with 3.5 cubic mm voxel size. Processed scans were then individually inspected for each participant, for any visible distortions due to motion artifacts, post co-registration.

Statistically significant whole-brain maps of the activation patterns for each of the 4 conditions/regressors in the IGT, was modelled using *3dDeconvolve* in AFNI. The mean t-statistics output for each subject served as the input for analyzing group effects (*3dMEMA*) for PPS, HMC groups (Fig 1), case studies (S1 Fig); as well as the effect of co-variates (Analysis of Co-variance-ANCOVA) such as mental health status (Fig 2), antipsychotic medication dosage (Fig 3) psychosis symptoms (Fig 4), IGT score (Fig 5), and DOI (Fig 6) on brain activation maps. Brain activation maps in all figures are viewed on Montreal Neurological Institute (MNI) standard stereotaxic space [48].

For the ANCOVA on medication dosage (Fig 3), we first converted first and second generation antipsychotic medications prescribed to PPS, to a common baseline of Haldol equivalents to ensure parity across brand and type of medication [49]. We then calculated the lifetime prescription dosage and the dosage on day of scan for each PPS on this common baseline.

In order to examine real world effects of decision-making quality in both groups, we administered the Decision Outcome Inventory (DOI) [50], a self-report survey of avoidance of negative outcomes of one’s real-life decision-making.

T-test analyses were done to compare the PPS and HMC groups on their performance on the DOI, including the IGT score. Along with these instruments, PPS were also interviewed and rated on their positive and negative psychosis symptoms using the Positive and Negative Psychosis Syndrome Scale [51] (PANSS) by trained research personnel, on the day of scan.

## Supporting information

S1 File (DOCX)

S1 Table FMRI activation patterns. (DOCX)

S1 Data (XLSX)

S1 Fig (TIF)
