# Supplementary material for: Patients on the psychosis spectrum employ an alternate brain network to engage in complex decision-making
Source: PLoS One. 2020 Sep 11;15(9):e0238774. doi: 10.1371/journal.pone.0238774 (PMC7485831; doi:10.1371/journal.pone.0238774)
Supplement: S1 File — (DOCX) [file pone.0238774.s001.docx]

**Case Studies:** Patient A with a diagnosis of schizophrenia affective disorder (SAD) is a high functioning individual, antipsychotic medication free for 5 years (by personal choice) at the time of testing and has been studied in another research protocol[1]. Patient B with a diagnosis of schizophrenia (SZ) has been antipsychotic medication free for a decade by personal choice. We examined their individual BOLD response on the IGT conditions (Figure S1). During card selection (Choose ), Patient A (SAD) shows significant activation in the brain reward circuit ranging from the cerebellum, through the midbrain DA areas and ventral striatum as well as bi-Ins (Figure S1, Panel A), a key node of the salience network. However, there is no activation of the mPFC, with significant activation of the l-dlFC node in Choose and Lose conditions, with bi-dlPFC (Win, Learning/Updating) and bi-SPL nodes of DAN in the Learning/Updating conditions (Figure S1, Panel A). Patient B (SZ) has no significant brain reward circuit activation (Figure S1, Panel B) in any of the IGT conditions. On the other hand, Patient A displays sub-cortical activation of brain reward circuitry with hypo frontality of the mPFC node of this circuit. This suggests that dysregulation of the brain reward circuit may be due to the condition of psychosis symptoms associated with psychiatric diagnoses of PPS, rather than the dopaminergic antagonist effects of antipsychotic medication. It is of note that patient B (SZ) self-reported strong auditory and visual hallucinations as a part of her daily life. This may be consistent with the significant activations in the visual association cortex (including fusiform gyrus) and Heschyl’s gyrus for this subject (Figure S1).

**Figure S1: Case studies of functional neuroimaging brain activation patterns on the Iowa Gambling Task in two medication-free psychosis spectrum patients**

**………………………. Insert figure S1 here ……………………….**

*Patient diagnosed with schizoaffective disorder (SAD-Panel A), and with schizophrenia (SD- Panel B). Left to right (within Panels A and B) are the axial, coronal and sagittal views of the Blood Oxygenation dependent (BOLD) response on each of the four task conditions of the Iowa Gambling Task (IGT).*

1. Palmer CM, Gilbert-Jaramillo J, Westman EC. The ketogenic diet and remission of psychotic symptoms in schizophrenia: Two case studies. Schizophrenia research. 2019;208:439.
