# Supplementary material for: Patients on the psychosis spectrum employ an alternate brain network to engage in complex decision-making
Source: PLoS One. 2020 Sep 11;15(9):e0238774. doi: 10.1371/journal.pone.0238774 (PMC7485831; doi:10.1371/journal.pone.0238774)
Supplement: S1 Table — (DOCX) [file pone.0238774.s002.docx]

**Table S1: FMRI Activation Patterns**

| **IGT Task Condition** | **Figure 1: IGT task** | | **Figure 2: IGT task in PPS relative to HMC** | |
| --- | --- | --- | --- | --- |
|  | ***PPS*** | ***HMC*** |  | |
| ***Choose*** | DAN, l- AC, l-Ins | BRC, l-dlPFC, mi-TG | SPL/precuneus, mPFC | |
| ***Lose*** | Mid-brain DA dlFC, TG, FG, mPFC | Mid-brain DA, r-OFC, TG, | TG, FG, Mid-brain DA, OC, anterior r-Ins, r-OFC, bi-SPL, mPFC, posterior cingulate | |
| ***Win*** | Ins, mPFC, r-OFC,TG, l-FG | Mid-brain DA, r-Ins, r-OFC, TG, striatum, DAN, CE | Mid-brain DA, TG, r-dlPFC, Ce, SN striatum, | |
| ***Learning/Updating*** | DAN, pHG | l-OFC, Ce, OC, dlPFC | DAN, CE, OC, striatum, dorsal Acc, Mid-brain DA area | |
|  | **Figure 3: IGT task co-varied with antipsychotic medication dosage in PPS (high versus low)** | | **Figure 4: IGT task co-varied with total psychosis symptoms in PPS (high versus low)** | |
|  | ***Dose Years*** | ***Daily Dose*** |  | |
| ***Choose*** | SPL/precuneus, l-AC, PFC | SPL/precuneus, l-AC, PFC | Ins, mPFC | |
| ***Lose*** | dlPFC, mid-brain DA, FG, AC, TG, striatum, mPFC | mid-brain DA, AC, TG, striatum, mPFC | Mid-brain DA, FG, dlPFC, AC, mPFC | |
| ***Win*** | AC, TG, DAN | AC, TG, DAN, Ins, striatum | Mid-brain DA, FG, AC, mPFC, DAN | |
| ***Learning/Updating*** | DAN, pHG, FG, OC | DAN, pHG, OC | DAN, AC, pHG, ventral striatum | |
|  | **Figure 5: IGT task co-varied with IGT score (high versus low)** | | **Figure 6: IGT task co-varied with real-life DOI** | |
|  | ***PPS*** | ***HMC*** | ***PPS*** | ***HMC*** |
| ***Choose*** | l- AC, l-Ins, l-SPL, mFC | BRC, dlPFC, TG, FG, pHG | l- AC, l-Ins, | BRC, TG, pHG |
| ***Lose*** | DAN, Mid-brain DA, AC, TG, anterior l-Ins, mPFC | Mid-brain DA, r-OFC, TG, OC | DAN, Mid-brain DA, AC, TG, FG, r-OFC anterior r-Ins, mFC, m-precuneus, CE, posterior cingulate, ventral striatum | BRC, TG, OC, FG |
| ***Win*** | TG, l-FG, Ins, mPFC, r-OFC, | Mid-brain DA, r-Ins, r-OFC, TG, striatum, DAN, CE | TG, l-FG, AC, dlFC, Ins, mPFC, | Mid-brain DA, anterior, SN, TG, l-dlPFC, CE, striatum |
| ***Learning/Updating*** | DAN, r-mPFC | DAN, l-OFC, Ce, OC, dlFC | Ventral striatum, pHG, DAN, mPFC, OC | l-OFC, Ce, OC, FG, r-globus pallidus |
|  | **Figure S1: IGT task in 2 medication-free PPS (case studies)** | | ***Legend***:  Red: activation/high ; blue: de-activation/low  Bilateral (bi), Left (l); Right (r).  **Brain reward circuit** (BRC): cerebellum (Ce), mid-brain dopamine area (mid-brain DA), thalamus, striatum, medial prefrontal cortex (mPFC).  **Dorsal attentional network** (DAN): dorsolateral PFC(dlPFC); superior parietal lobule (SPL)/precuneus  **Salience Network** (SN): Insula, dorsal anterior cingulate (Acc) or mPFC  Insula: Ins  **Temporal gyri**: TG (middle-mi, superior-s, lower-lo); auditory cortex: AC  orbital frontal cortex: OFC  Para hippocampal gyrus: pHG  Occipital Cortex: OC  Fusiform Gyrus: FG | |
|  | ***Patient-SAD*** | ***Patient -SZ*** |  |  |
| ***Choose*** | Ce, midbrain DA, ventral striatum, Ins, mPFC | l-AC,STG |  |  |
| ***Lose*** | AC, mi-TG, l-dlFC, Ce, SPL | Ce, OC, FG, AC |  |  |
| ***Win*** | AC, s-TG, l-dlFC, | r-AC |  |  |
| ***Learning/Updating*** | DAN, Ce | No activation |  |  |
