# Supplementary figures and images for: Patients on the psychosis spectrum employ an alternate brain network to engage in complex decision-making
Source: PLoS One. 2020 Sep 11;15(9):e0238774. doi: 10.1371/journal.pone.0238774 (PMC7485831; doi:10.1371/journal.pone.0238774)

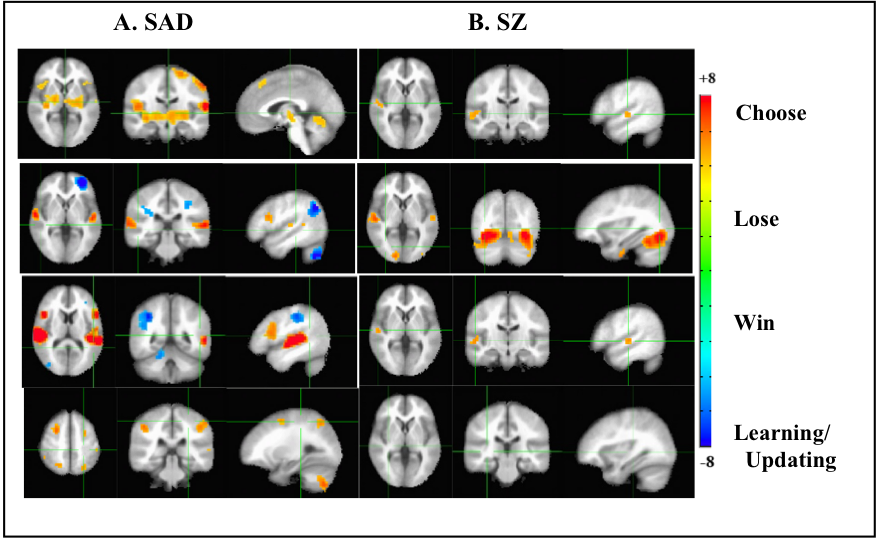

Supplement: S1 Fig — (TIF) [file pone.0238774.s004.tif]
